# Experimental FIA Methodology Using Clock and Control Signal Modifications under Power Supply and Temperature Variations

**DOI:** 10.3390/s21227596

**Published:** 2021-11-16

**Authors:** Francisco Eugenio Potestad-Ordóñez, Erica Tena-Sánchez, José Miguel Mora-Gutiérrez, Manuel Valencia-Barrero, Carlos Jesús Jiménez-Fernández

**Affiliations:** 1 Department of Electronic Technology, University of Seville, 41004 Sevilla, Spain; erica@imse-cnm.csic.es (E.T.-S.); manolov@imse-cnm.csic.es (M.V.-B.); cjesus@imse-cnm.csic.es (C.J.J.-F.); 2 Microelectronic Institute of Seville (IMSE-CNM-CSIC/US), 41092 Sevilla, Spain; jmiguel@imse-cnm.csic.es

**Keywords:** experimental fault attack, IoT, power supply variation, temperature variation, ASIC, vulnerability, stream cipher

## Abstract

The security of cryptocircuits is determined not only for their mathematical formulation, but for their physical implementation. The so-called fault injection attacks, where an attacker inserts faults during the operation of the cipher to obtain a malfunction to reveal secret information, pose a serious threat for security. These attacks are also used by designers as a vehicle to detect security flaws and then protect the circuits against these kinds of attacks. In this paper, two different attack methodologies are presented based on inserting faults through the clock signal or the control signal. The optimization of the attacks is evaluated under supply voltage and temperature variation, experimentally determining the feasibility through the evaluation of different Trivium versions in 90 nm ASIC technology implementations, also considering different routing alternatives. The results show that it is possible to inject effective faults with both methodologies, improving fault efficiency if the power supply voltage decreases, which requires only half the frequency of the short pulse inserted into the clock signal to obtain a fault. The clock signal modification methodology can be extended to other NLFSR-based cryptocircuits and the control signal-based methodology can be applied to both block and stream ciphers.

## 1. Introduction

The new cryptosystems have been proven to be mathematically safe, since it would take a large amount of time and computing resources to mathematically compromise their safety. However, there are new analysis techniques that do not attack the mathematical implementation of the algorithm itself, but the physical implementation of it. These analysis techniques are known as passive and active attacks [1,2,3,4,5,6,7]. Passive attacks are the so-called side channel attacks, which exploit physical leakages during encryption processes, such as power consumption, electromagnetic radiation, or timing, to reveal secret information, e.g., power analysis (PA) [1,2]. On the other hand, active attacks are any invasive or non-invasive attacks that exploit the information provided by cryptographic devices during an erroneous encryption or decryption process. In our case, we focus on active non-invasive attacks that, together with the fault analysis (FA) [3,4,5,6,7], are able to compromise the security of cryptosystems without causing any damage to the circuit or evidence of manipulation. With this technique, if an attacker is able to inject transient faults into the encryption or decryption process and mathematically compare the correct and faulty outputs of the cipher, the attacker would be able to recover the secret key.

The continuous evolution of new ways to compromise the security of cryptocircuits in order to access sensitive data has attracted the attention of the international community. This has led new designers to use attacks as a vehicle to analyze the vulnerabilities of new cryptosystems and already standardized ones. Taking on the role of the attacker and compromising the security of the cryptosystems allow designers to determine the vulnerabilities of the cryptosystems, thus allowing them to select the most appropriate guidelines and countermeasures to try to minimize the detected security flaws.

In this paper, different attack methodologies are presented with the aim to test the vulnerabilities of different cryptocircuits in a real scenario. These methodologies are within the active, non-invasive attack group due to the fact that we do not modify the implementation of the cryptocircuits. The attacks consist of injecting faults into the ciphers under analysis using different techniques and combining them with the modification of the power supply and temperature. In our case study, we consider the Trivium cipher [8] and its different versions for power reduction and multi-radix outputs [9].

The Trivium cipher was an eSTREAM project finalist and it was selected as an ISO Standard (ISO/IEC 29192) [10]. It is a synchronous stream cipher that uses an 80 bit secret key (K) and an 80 bit initial value (IV), generating up to $2^{64}$ bits of key stream. The hardware implementation of Trivium consists of three circularly arranged non-linear feedback shift registers (NLFSR), containing 288 bit internal states. It was designed to have the most simplified structure without sacrificing security, speed, or flexibility, making it an ideal candidate for Internet of Things (IoT) applications, where resource and energy constraints play a major role.

Since a fault injection attack needs the theoretical formulation of the FA and vice versa, in order to study if the attack methodologies presented are useful or not, it is necessary to know the FAs carried out over the Trivium cipher. The first differential fault analysis (DFA) of Trivium was presented by Hojsík and Rudolf [11], where two different mathematical formulations were exposed. The second technique is the most efficient one, allowing the secret information to be retrieved with an average of 43 fault injections. In [12], the authors assume less restrictive assumptions than those taken in [11] and they are able to retrieve the secret information with an average of 3.7 fault injections. In [13], the authors affirm that it is possible to break the Trivium with only one fault injection. In [14], an improvement over the system constraints of [12] is presented. Their method allows the cipher to be attacked using different fault models, injecting a fault into an unknown clock cycle and retrieving the secret information with four fault injections. The main assumption of these works and, in general, to perform a DFA of the Trivium cipher is that, in order to retrieve the secret information, it is necessary to inject only one faulty bit over any of the 288 bits of the internal state of the cipher. This assumption establishes that, if an attacker is able to achieve this kind of injection, the cipher could be endangered with the theoretical DFA. It should be noted that none of these works attempt to experimentally test the vulnerability of the Trivium cipher, proving the feasibility of the faults needed, but rather base their hypotheses on theoretical assumptions, discarding the experimental aspect. On the other hand, good examples of the continuous and recent interest in breaking this encryption algorithm, apart from the DFA, are the works presented in [15,16,17,18,19,20]. An attack on the bitstream used to configure an FPGA with the Trivium cipher implemented is presented in [15]. The authors propose an attack by changing three LUTs to reduce the non-linearity of the cipher. In [16], the authors present a key recovery attack on Trivium based on the nullification technique of the Boolean polynomial and reducing the output polynomial of 855-round Trivium. In [17], the authors present a guess-and-determine attack on Trivium, where they propose an improvement in order to increase the number of attack scenarios based on the combination of time-memory-data trade-offs and take advantage of quadratic conditions. In [18], a conditional differential attack applied to the Trivium cipher is presented. The authors perform key recovery attacks on the 978 round, detecting non-randomness up to the 1108 round. With this, the authors offer an improvement to attackers implementing differential attacks. A study of cubic attacks against the cipher is proposed in [19] and a new method for finding non-linear superpolies using the linearity test principle is also described. Finally, in [20], a cube attack is performed, showing that it is able to recover the key in the 781 round of Trivium.

On the other hand, the experimental analysis to test the possibilities of injecting faults experimentally into the cipher was studied in [21,22]. In [21], the authors showed that it is possible to inject faults into the cipher when it is implemented in FPGA. Nevertheless, in these works, the attack systems are internally generated in the FPGA; that is, the cipher is implemented together with the attack system. With this, signal filtering or synchronization problems are eliminated, allowing the experimental tests to move away from a real scenario where an attacker only has access to the input and output pads of the circuit that implements the cipher and cannot control anything else. In [22], the authors show how to recover secret information from the cipher in a model closer to a real scenario than that applied in FPGA. For this purpose, they use a standard Trivium cipher implemented in an application-specific integrated circuit (ASIC) and perform external clock attacks, which, combined with a DFA model, are able to recover the secret key. However, this work only includes the analysis of the standard cipher and does not study the possibility of using the methodologies presented in this paper, especially those concerning the fault injection through the control signals. The strongest point of the present paper is the proposed methodology to inject faults via the control signals, together with the experimental validation of this method, which is, to the best of our knowledge, the first time this has been presented in the literature, showing excellent results.

The main objectives of this work are the following: (i) different attack methodologies in order to study the vulnerability of different cryptocircuits, inserting short pulses into the clock signal, or modifying the control signals; (ii) the optimization of fault injection techniques using supply voltage and temperature variations; (iii) experimental verification of the proposed methodologies; (iv) evaluation of different Trivium versions also considering the effect of different routings for the same implementations; (v) experimental setup and attack on different designs of Trivium proposals implemented in an ASIC. In our case study, we considere different versions of the Trivium cipher in the closest way to a real scenario, where the attacker only has access to the input and output pads. For this purpose, different attack systems to inject faults externally were designed and applied in an ASIC circuit.

The rest of the paper is organized as follows: In Section 2, the ASIC design is described and the ciphers used for the test are presented. In Section 3, the attack methodologies and test conditions are described. Section 4 presents the experimental attacks using the different systems implemented for this work, the values and conditions selected for the test and the challenges faced. Section 5 describes the results obtained from applying the attack systems to the ciphers. Finally, in Section 6, the conclusions of this work are presented.

## 2. Description of the ASIC Used to Test the Proposed Attack Systems

**Figure 1:**
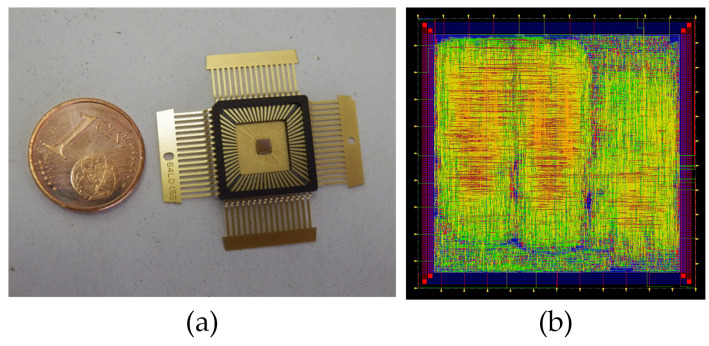
(**a**) ASIC; (**b**) ASIC’s layout.

In order to test and evaluate the efficiency of the proposed attack systems and to analyze the influence of the attacks on different implementations of the same cryptographic algorithm, a 90 nm Taiwan Semiconductor Manufacturing Company (ASIC) implementation was used [9]. It should be noted that, in this paper, we do not present the design of the ciphers, but use them to evaluate the attack systems and to study the vulnerability of each cipher with different implementation approaches against these types of attacks. In Figure 1a, a picture of the ASIC circuit is shown and, in Figure 1b, the core ASIC layout is presented. This ASIC was designed with different purposes, but, in this paper, it was used as a demonstrator vehicle. This led us to be close to the real scenario due to the fact that the cryptocircuits were implemented in the ASIC and we only had access to the inputs and outputs of the chip. Different versions of the Trivium stream cipher were implemented in this ASIC. These versions are the standard Trivium, the multi-radix Trivium (with 2, 8 and 16 bits of key stream for each clock cycle); and the low-power consumption version of each one of these versions [9]. Additionally, it is noteworthy to notice that we had two versions of each one with different routings, which is the effect of different routings also evaluated in this paper. In total, we had 8 different versions of the Trivium stream cipher, 16 in total, considering those with alternative routings. All of them were loaded with the key and the IV serially from outside the ASIC and the clock and control signals of the Triviums were connected directly to the core pads. Through a selection signal, the Trivium version under analysis was selected. A brief description of each version of the Trivium cipher is presented below. These descriptions allow us to perform a simplified analysis of the response of each cipher to the presented attack systems. For further information, please refer to the paper in which they are presented [9].

### 2.1. Standard Trivium

**Figure 2:**
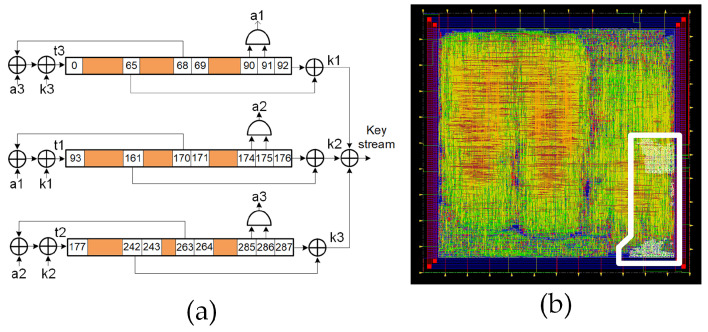
(**a**) Schematic representation of the standard Trivium stream cipher; (**b**) layout of the ASIC highlighting the location of the two standard Triviums.

The Trivium stream cipher is a synchronous cipher designed to generate up to $2^{64}$ bits of key stream from an 80 bit secret key and an 80 bit IV. The cipher architecture is based on three shift registers comprising 288 bits in total, as well as combinational logic to provide feedback. This feedback is those flip-flops denoted by the positions 0, 93 and 177. Similar to other synchronous stream ciphers, the underlying algorithm needs to be initialized with the load of 288 bits into the shift register (internal state) comprising one secret key, one IV and a stream of zeros and ones. Before generating a valid key stream, the cipher needs to run for 1152 clock cycles. From that moment on, it generates a valid pseudorandom bit sequence. Figure 2a shows the schematic of the Trivium’s internal structure and Figure 2b shows its layout, which is highlighted by the location of the two standard Trivium instances.

### 2.2. Multi-Radix Trivium

**Figure 3:**
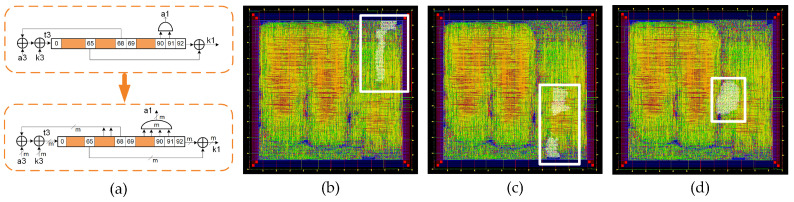
(**a**) Schematic representation of the first register for the standard cipher and multi-radix cipher (top and bottom, respectively). (**b**) Layout of the ASIC highlighting the location of the multi-radix ×2 layout, (**c**) multi-radix ×8 layout and (**d**) multi-radix ×16 layout.

In addition to the standard version of Trivium, it is possible to build different versions of this cipher, which is able to generate more than one bit of key stream per clock cycle [8]. These versions are known as multi-radix Triviums. The internal state maintains the same number of bits, but additional logic for the feedback is added. Depending on the version, it is possible to achieve up to 64 bits of key stream per clock cycle. In our case, the multi-radix ×2, multi-radix ×8 and multi-radix ×16 are considered. In Figure 3a, a comparison between the schematic representation of the standard Trivium first register and the multi-radix version is shown. The *m* denotes the number of bits per line to perform the ×2, ×8 and ×16 versions. In Figure 3b–d, the multi-radix ASIC layout highlighting the location of the ×2/x8/x16 versions, respectively, is shown.

### 2.3. Low-Power Trivium

**Figure 4:**
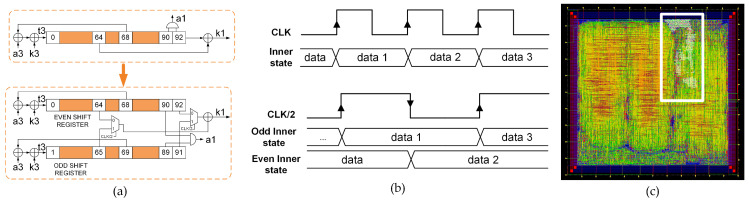
(**a**) Schematic representation of the power reduction technique for Trivium. (**b**) Waveforms for standard and low power Trivium. (**c**) Layout of the ASIC highlighting the location of the low power Trivium.

Through the parallelization of the shift register technique, a power reduction was achieved. Figure 4a shows a schematic representation of the power reduction technique for Trivium; at the top, the first partial register of the standard Trivium from Figure 2 is displayed, while, at the bottom, the parallelization of the partial shift registers for power reduction is shown. Figure 4b shows the clock and data waveforms for each version and Figure 4c shows the layout for this cipher version. This technique consists of dividing the shift register into two, each of which is half the length of the original. These registers are called even and odd registers, as shown in Figure 4a. Both registers, the even and odd registers, are triggered by an internal clock, which has half of the input clock frequency; however, from a practical point of view, the complete shift register has the same frequency as the standard (Figure 4b). Due to the fact that only half of the data are shifted in each half clock cycle, a power reduction is achieved. This technique achieves a significant reduction in power consumption, but requires additional logic by modifying the original implementation in the feedback. In this case, the feedback positions are the flip-flops denoted by positions 0, 1, 93, 94, 177 and 178.

### 2.4. Multi-Radix Low-Power Trivium

**Figure 5:**
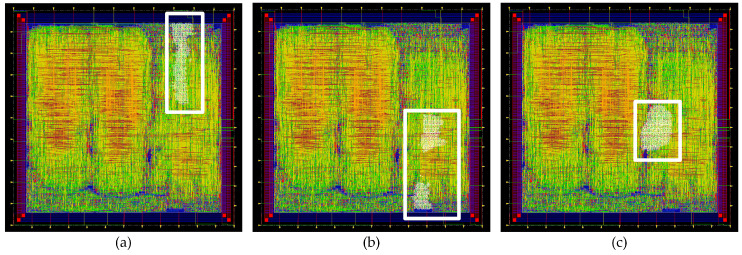
Layout of the ASIC highlighting the location of the (**a**) multi-radix ×2 low power Trivium, (**b**) multi-radix ×8 low power Trivium and (**c**) multi-radix ×16 low power Trivium.

As in the low-power version of the standard Trivium, the same technique was applied for the multi-radix versions of Trivium. With this, it is possible to generate multiple bits of key stream with minimum power consumption per clock cycle. As in the previous implementation, the feedback positions are the flip-flops denoted by the positions 0, 1, 93, 94, 177 and 178. It is noteworthy that, if the power reduction technique or the multi-radix output technique is applied, it is necessary to add more logic. Joining both in the same design means adding considerable logic. As already stated, we considered the ×2, ×8 and ×16 low-power versions for the vulnerability assessment. The locations of each of the multi-radix low-power versions of Trivium are highlighted in Figure 5.

## 3. Attack Methodologies

In order to test the vulnerabilities of the different stream ciphers implemented in the ASIC technology, two different fault injection systems were designed and used. The first system uses the technique of injecting small pulses into the clock line [3], which is combined with the supply voltage and circuit temperature variations. The second system manipulates the control signals of the circuit. Both techniques are explained in detail in the following subsections.

### 3.1. Attack Using Clock Glitches

**Figure 6:**
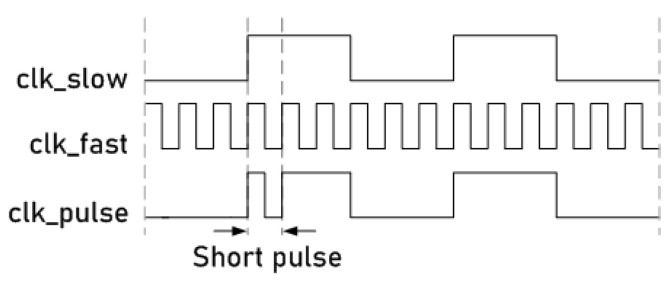
Waveform representation of the short pulse insertion in a clock signal.

This attack system uses an active non-permanent and non-invasive attack that consists of manipulating the system’s clock signal [3]. Figure 6 shows a general schematic representation of this technique. In this technique, two clock signals are combined in a specific clock cycle using a low and a high frequency, respectively, to obtain a short pulse in the main clock line of the circuit. In Figure 6, each of these is denoted as clk_slow (slow clock signal), clk_fast (fast clock signal) and clk_pulse (main clock signal applied to the cryptographic circuit).

Using this technique it is possible to inject transient faults in the circuits due to the fact that the clock restrictions of the flip-flops are violated. In the case of crypto-circuits, the time violations are translated to fault injections in the shift registers composed by the flip-flops; therefore, this allows this technique to be used in any NLSFR-based ciphers. In our case, for the Trivium cipher, the faults were injected into the internal state composed by the three partial shift registers.

The use of this technique brings with it a certain degree of experimental difficulties in injecting faults into the crypto circuit. It should be noted that the Trivium stream ciphers can operate at high frequencies. Therefore, to introduce a fault into them, it is necessary to achieve an even higher frequency. This presents a problem, since, unlike the circuit core, the input and output pads of ASICs do not support very high frequency signals; therefore, high-frequency signals, e.g., small pulses, can be filtered out. We observed, through numerous tests, that too-high frequencies are filtered out by the circuit pads or inject many faults at the same time.

During the tests carried out on ASIC implementation, it was observed that, if a single clock pulse was induced, it was not possible to inject faults into the circuit. However, if two consecutive clock pulses were induced, it was possible to inject faults. This could be due to different factors; for example, a clock signal is not, in fact, an absolute logical value—0 or 1—but a continuous voltage signal that varies between two maximum and minimum values. Voltage changes in the clock signal for the high frequencies that are needed to inject faults may not stabilize at the logic 0 or 1 (logic value change); therefore, the circuit may not consider that situation as a change in the clock signal. Therefore, by using two small clock pulses consecutively, it is possible for at least one of them to be considered by the circuit as a change in the clock signal, allowing faults to be injected. In the following, we discuss the introduction of a small clock pulse for simplicity, but it must be taken into account that, in fact, two consecutive clock pulses were used due to the above-described phenomenon.

The clock signal manipulation technique has been proven to be very useful when applied to the Trivium cipher implemented in FPGAs [21]. In these systems in which FPGAs were used, the pulse injection system was internally generated in the FPGA itself. Thus, there was no problem in filtering the short pulses or synchronizing signals that were too high. In our case, the attack system for signal and clock pulse generation was externally implemented, since the device under attack was an ASIC implementation. This caused great difficulty in designing a system capable of finding the right fault frequency that was able to inject faults into the system.

**Figure 7:**
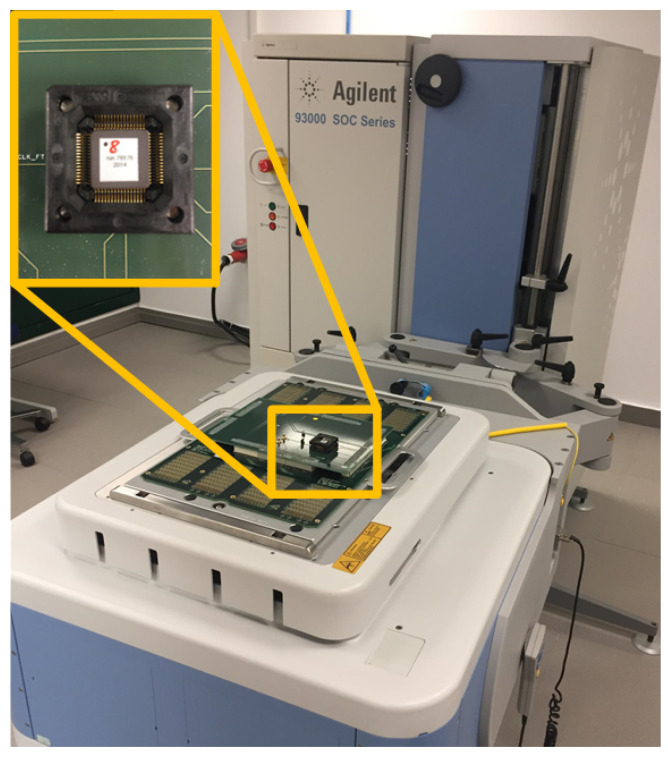
Experimental setup with Agilent 93000 equipment and ASIC implementation.

**Figure 8:**
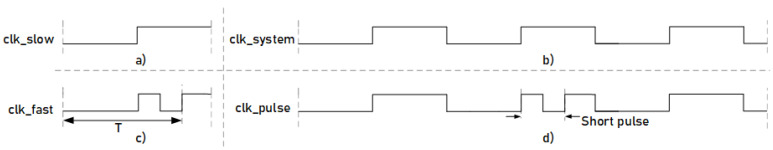
Representation of the waveforms generated to setup the short pulse insertion. (**a**) One period of the slow clk; (**b**) continued use of the slow clk waveform to perform the system clk; (**c**) one period of the fast clk; (**d**) combination of the system clk and fast clk to obtain the short pulse in the system clk.

To set up the attack system, we used the Agilent 93000 instrument; the experimental setup is shown in Figure 7. In this figure, the ASIC circuit connected to a specific board, which allows the pin connection between the 93000 tool and the ASIC circuit to be establish, is shown. Agilent 93000 is powerful equipment that allows us to have a great control over the generation of signals, to define different waveforms and to combine them. Note that we used this tool as we had access to it, but the attack system can also be mounted using cheaper and commercial tools. This tool is controlled by Agilent software, which allows users to define a multitude of variables. Among them, it is possible to define input signals of the circuit to introduce the stimulus, output signals for data reading and supply voltage and ground. The different waveforms are defined using waveform windows. With these windows, the clock signals and input data are defined. The windows have a programmable number of edges, from 1 to 8, configured as rising or falling edges. Figure 8 shows the different waveform windows that were defined and used with Agilent 93000. Figure 8a represents a waveform window where the configuration is one of the eight programmable edges programmed as the rising edge at 50% of the window. Figure 8c represents a waveform window where the configuration is three of the eight programmable edges programmed as rising, falling and rising edge. Having defined these two waveforms, if they are used repeatedly, a clock signal can be obtained, as shown in Figure 8b, where waveform (a) is repeated three times. On the other hand, if waveform (a) and (c) are combined, a clock signal can be obtained with the injection of a small pulse, as shown in Figure 8d, where waveform (a) is repeated twice and waveform (c) once. Waveform (b) is the clk_system and waveform (d) is the clk_pulse. These clock signals are connected to the circuit to carry out the attacks by modifying the clock signal.

**Figure 9:**
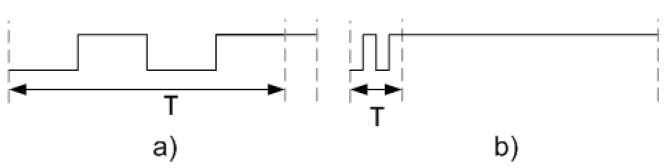
Representation of the short pulse definition using the variable T. (**a**) Short pulse with a high period and (**b**) short pulse with a small period.

To determine the appropriate attack frequency to introduce faults in the ciphers, it is necessary to modify the waveform (c) dynamically, i.e., using a smaller or larger small pulse. To modify the waveform (c), the tool allows us to define a new variable denoted as *T*, whose value can be modified. Figure 8 shows the use of the variable *T* when a waveform is defined. Through the modification of this variable, it is possible to generate the short pulse with a smaller or greater period—Figure 9a,b, respectively. This characteristic allows the fault frequency that is necessary to inject faults into the ciphers to be found. Once the value of T, which defines the period of the small clock pulse that allows faults injections to happen, is defined, this waveform configuration is used to inject the small pulse into the desired clock cycle to attack the ciphers.

### 3.2. Combining Clock Attacks with Power Supply Variations

In this case, the previously described attacks using the short pulse in the clock signal were combined with the modification of the circuit supply voltage. As described in the literature, the underpowering of the circuit power supply causes frequency degradation and even a malfunction [3,23]. Decreasing the supply voltage in CMOS circuits causes them to operate slower, i.e., their maximum operating frequency drops considerably and operating times become longer. Thanks to this decrease in the maximum operating frequency, the periods of clock pulses required to inject faults increase, facilitating attacks. In this regard, Agilent 93000 allows us to modify the supply voltage of the circuit core and/or the ring pads of the circuit. With this, it is possible to supply the ring pads of the circuit with the nominal voltage but, at the same time, to modify the voltage of the core, using a low or a high supply voltage. This can be carried out as, in an ASIC circuit, the ring supply voltage and the core supply voltage are implemented in different pins. This makes it possible to analyze whether the modification of the supply voltage makes it easier to inject faults when a pulse is applied to the clock signal. In Table 1, the values of the core power supply recommended by the manufacturer and the values used in the tests are shown. As shown in the table, both the maximum and minimum voltages are out of the range guaranteed by the manufacturer of the ASIC circuit. The aim of using these values is to cause the circuit to operate outside its ideal operating range to check if the degradation of the operating frequency helps to inject faults through the clock signal. It is important to note that we ensured that the underpowering used in this experimental setup did not inject faults and was thus used to achieve an easiest fault injection, meaning that the faults are caused by the insertion of the short pulse in the clock signal.

### 3.3. Combining Clock Attacks with Temperature Variations

The effect of temperature on the operation of CMOS-based electronic circuits has been extensively studied in the literature [24,25]. Temperature variations affect, among other factors, the maximum operating frequency of the circuit. As the temperature increases, without causing irreversible damage, the maximum operating frequency of the circuit is degraded. On the other hand, at low temperatures, the maximum operating frequency of the circuit tends to be optimal. In this case, the clock signal attacks were combined with the modification of the circuit’s operating temperature. For this purpose, the instrument Benchtop Precision Temperature Forcing System Thermonics T-2650 BV was used in combination with the small pulse insertion performed by Agilent 93000. With this tool, it is possible to modify the circuit temperature from −55 to 200 °C. Table 2 shows the values of the temperature recommended by the manufacturer and the values used for the attacks.

It should be noted that, although the equipment allows the circuit to be subjected to a much higher or lower temperature and that the manufacturer guarantees the correct operation at a higher or lower temperature, we selected these values as a higher temperature can burn the board where the ASIC circuit is implemented and a lower temperature can create ice and produce irreversible damage. Therefore, the values used are the maximum values that can be applied to study the behavior of the cipher under temperature changes without producing irreversible damage. It is noteworthy that, as in the case of the power supply, the temperature variation does not inject faults into the cryptocircuit.

### 3.4. Attack Using Control Signals

**Figure 10:**
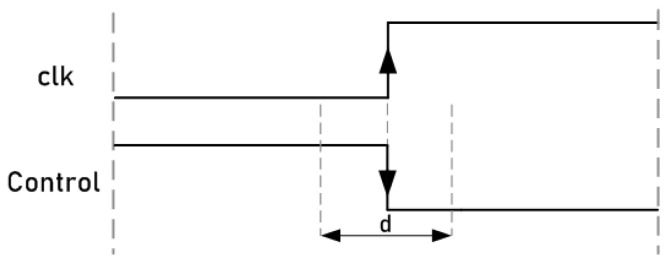
Representation of control signal manipulation.

This attack system uses a technique that consists of manipulating the control signals of the ciphers to inject faults into their internal registers. Unlike clock signals, circuit control signals do not have to be routed in a balanced way using special propagation lines to ensure minimum delay and skew. Since these signals are responsible for activating or stopping the operation of the ciphers, by activating the change in the value of the input signal of the flip-flops near to the active clock edge, it is possible to introduce faults in the registers through malicious manipulation. Figure 10 shows a representation of the control signal manipulation. Similar to the clock signal attacks, a waveform window was defined in the 93000 tool for the control signal. In this waveform, a falling edge was defined for the control signal. In this case, the attacks consisted of positioning the falling edge of the control signals close enough to the active clock edge of the ciphers to cause malfunction and achieve fault injections due to the setup and hold time violations. As shown in the figure, as a general example, there is a range denoted by *d*, near the active edge of the clock, where, if the control signal changes, it could produce errors. Fault injections may occur due to the fact that, if the change in the control signals occurs too close to the active edge of the clock signal of the flip-flops, it causes violations in the setup times of the flip-flops. These timing violations cause the flip-flops to set a new value or keep their previous one, forcing incorrectly sampled values during their transitions. On the contrary, out of the *d* range, if the control signal changes, no errors occur. The values of this range are described in the following section.

In contrast to the case of clock signal attacks, through this methodology, it is possible to inject faults into intermediate positions between flip-flops without logic between them. This is a great advantage over the clock signal attack methodology as, without carrying out invasive attacks, it is possible to insert faults in any position, not only in those with a greater clock delay. This allows this technique to be used not only in NLSFR-based ciphers but also in any other types of ciphers, i.e., block or stream ciphers.

## 4. Experimental Attack

In order to test the attack systems and analyze the vulnerability of the ciphers implemented in ASIC technology, an attack plan was developed. The experimental attack was tested using an ASIC, where the different versions of the Trivium cipher were implemented. Each version of the Trivium ciphers was implemented in duplicate, that is, two identical ciphers implemented in parallel whose only difference is the routing. To carry out the test, the Agilent 93000 test tool and Thermonics T-2650 BV tool were used to attack the ciphers and modify the operating conditions of the circuit. The computer used to analyze the results was a Core-i5 desktop PC processor with 8 GB of RAM with the Matlab software.

**Figure 11:**
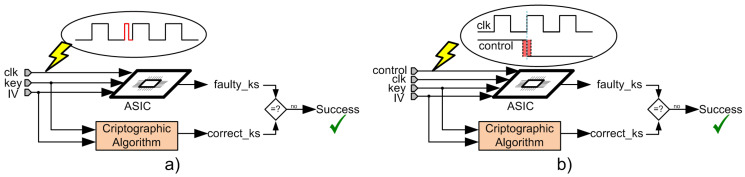
Schematic representation of the implemented attacks. (**a**) attack process manipulating the clock signal; (**b**) attack process manipulating the control signals.

A simplified schematic representation of the implemented attacks is shown in Figure 11a,b. It is noteworthy that Figure 11a represents both attacks manipulating the clock signal in combination with voltage variation and temperature variation, but, for simplicity, only one scheme is represented. In this figure, the cryptographic algorithm inside the box denotes the cipher under analysis that operates correctly and the ASIC circuit diagram denotes the same implemented cipher that is attacked, where faults should be inserted. As shown in Figure 11a, the clock signal of the ASIC circuit was manipulated by introducing a small clock pulse. On the other hand, in Figure 11b, instead of manipulating the clock signal, the control signals of the ciphers implemented in the ASIC were manipulated. In both scenarios, after performing the attacks, the faulty key stream (faulty_ks) was compared with the correct key stream (correct_ks) and, if both were different, the attack performed had inserted faults into the cipher; therefore, the attack was successful.

### 4.1. Attack Plan

**Figure 12:**
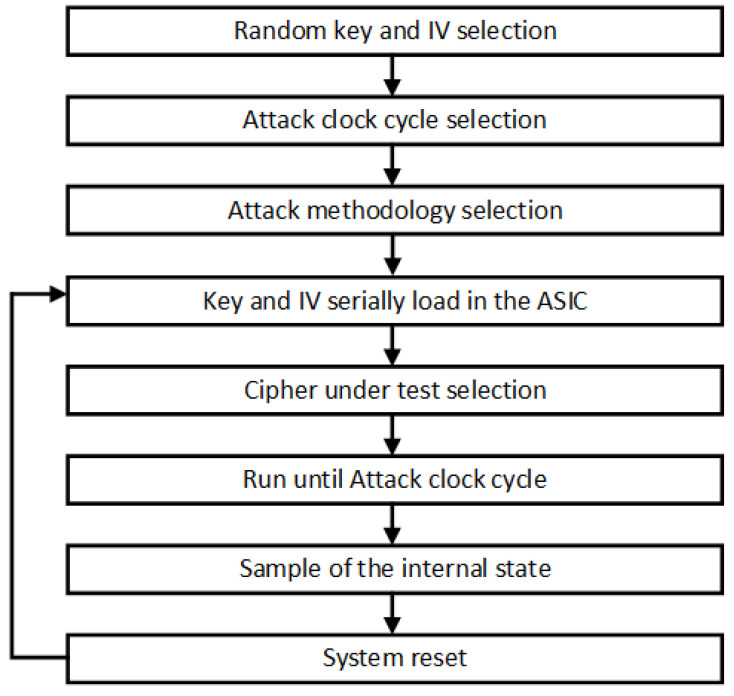
Representation of the attack process.

Two different clock signals were used, one for the load and the operation of the Triviums and another one for the data sampling at the circuit output. Figure 12 graphically shows the attack process step by step. To start the tests, a random key and IV were generated and a clock cycle was selected to carry out the attack. After this, an attack methodology was selected, fault insertion by clock signal or by control signal. Once the scenario was selected, the key and the IV were serially loaded in an intermediate register in the ASIC that is shared by all the ciphers. The cipher to be attacked was then selected and the value of the intermediate register was loaded in parallel into the internal register of the selected cipher. After the data load, a period of operation of the circuit was performed, up to 1152 clock cycles, which is the minimum time for the ciphers to produce a valid key stream. At this point, the correct internal state of the selected cipher was loaded in another internal register of the ASIC and serially sampled to an output with the second clock signal from a specific and known clock cycle (attack cycle previously selected). After that, the selected cipher was reset, the same key and IV was loaded and the process was repeated to carry out a new attack.

This process was carried out for each clock cycle selected for the attack. With these results, in each attack clock cycle, we obtained the correct and the faulty internal states, which allowed us to determine if any faults had been injected, as well as their number and position.

### 4.2. Selection of Experimental Value Ranges for Clock Short Pulse

In order to carry out the attacks, it was necessary to find the effective range of values for the short pulses used for the attacks. These ranges of values of the short clock pulses were used to determine the range of short clock pulses required to inject faults and determine the degree of difficulty in selecting the appropriate short clock pulse. As explained before, pulses that are too short can inject many faults and pulses that are too large do not inject any fault into the ciphers. For this reason, a study of the period range values was performed. This study consisted of testing different time periods for the short pulse to obtain the range of values where the successful fault injections were achieved. In Table 3, the results are shown. It is noteworthy that it was tested with minimum, nominal and maximum voltage values and with minimum, nominal and maximum temperature values. As shown in Table 3, instead of showing six different cases for the described operating conditions, only three cases are represented (Case 1, Case 2 and Case 3) for each one of the ciphers. Case 1 represents the obtained values for nominal supply voltage; Case 2 represents the obtained values for the minimum supply voltage; Case 3 represents the obtained values for the nominal temperature. Note that the other three cases, where the maximum supply voltage, minimum temperature and maximum temperature were applied, are not represented in the table. This is due to the fact that, if the maximum supply voltage is combined with the short pulse attack, the results are the same, as in the case of using the nominal supply voltage; therefore, it is only necessary to represent Case 1. In the case of the minimum and maximum temperature, the results are the same as in the case of using nominal temperature; therefore, it is only necessary to represent Case 3 as a result of the application of any temperature. Finally, as shown in Table 3, the obtained results for Case 1 (nominal supply voltage) and Case 3 (any temperature) are the same. This is due to the fact that the modification of the circuit temperature did not have an effect when a fault attack using short pulses was performed. It is noteworthy that these results are represented to highlight that the same results were obtained in Case 1 and Case 3. Therefore, only significant results obtained using nominal and minimum supply voltage are shown in the following section.

As shown in Table 3, Case 1, the results of the range values for the short clock pulses are very small, reaching only 100 ps, in the case of the multi-radix ×8 cipher, and not exceeding 3 ns. These results show the difficulty in achieving short clock pulses to inject faults due to the high precision required to generate the signals. On the other hand, if the results of the non-low-power designs of the ciphers are compared with the results of the low-power designs, it can be seen that, in the case of low-power versions, the range of values is higher than that in non-low-power versions. This is due to the fact that the low-power versions have more feedback and logic to perform the low-power reduction, facilitating the injection of faults into the internal state of the ciphers. In addition, if the non-multi-radix versions are compared with the multi-radix versions, the results are similar due to the addition of the logic to perform the multi-radix key stream output per clock cycle.

In the case of comparing the nominal and minimum supply voltage (Case 1 and Case 2), the results show that, if the attack using short pulses are combined with the underpowering technique, the value ranges of the short pulses needed to inject faults are doubled. Therefore, if the supply voltage reduction is applied, the fault injections become easier.

### 4.3. Selection of Experimental Value Ranges for Control Signals

**Figure 13:**
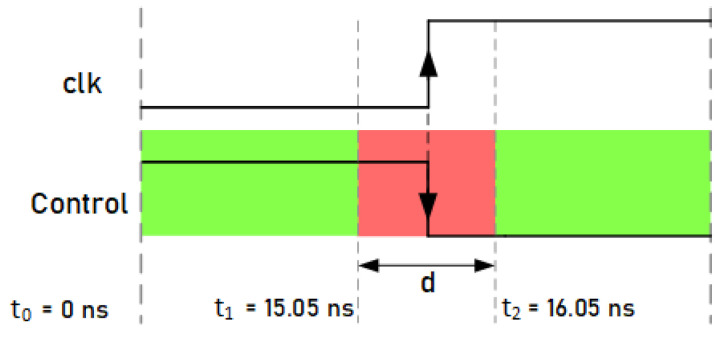
Representation of the range where the change in the control signal can cause fault injections.

In this case, the tests were only carried out on the standard Trivium cipher, since it is the one with the highest resistance to attacks. The aim of the test is to determine the limits of the range of values denoted by *d* ($t_{1}-t_{2}$), represented in Figure 13, that is, to establish the time range ($t_{1}-t_{2}$) near the active edge of the clock where, if the control signal changes, it injects faults. The values of *t* were experimentally obtained by sweeping the parameter *t*; this means assigning a specific time to the control signal’s falling edge and checking if any fault has been injected efficiently. After the experimental analysis, the results showed that faults can occur in the flip-flops of the cipher registers if the control signal changes between $t_{1}=15.05$ ns and $t_{2}=16.05$ ns. The value $t_{0}=0$ ns is the falling edge of the clock signal in this clock period. In the green zone, if the control signal changes, no faults occur. These results show the small margin that exists when introducing faults by manipulating the control signals, but, at the same time, if this margin is determined and the appropriate equipment is used, it is possible to inject faults.

## 5. Experimental Results

For simplicity, the tables show the data of the attack results related to five specific clock cycles where the attacks were carried out (using the same key and IV), specifying if faults were injected into each pair of Trivium ciphers. It is important to note that the other results that are not shown in this section, using other keys and IVs and different clock cycles for the attacks, display the same behavior as those presented in this section.

Each attack in each clock cycle was repeated one hundred times for each type of cipher with the same key and IV. Although injecting faults with the minimum supply voltage is easier than with the nominal supply voltage, the data used for the analysis of the positions where faults were injected and the analysis of the number of faults were obtained using the nominal supply voltage value. This ensured that the presented data, not having been obtained in a limit situation, can be extended to other implementations of Triviums in other technologies. On the other hand, since it was shown that temperature variations do not produce substantial variations in the results, all the data shown correspond to tests performed at nominal temperature (25 °C). Under these conditions, the widths of the pulses injected into the clock signal for each version of Trivium are those for Case 1 shown in Table 3.

The results are presented as follows. First, an analysis of the feedback flip-flop value transition in each of the attack cycles performed for the standard Trivium cipher using the same key and IV is described. Second, the results obtained after attacking the ciphers with the nominal supply voltage and using the insertion of a short pulse in the clock signal are presented, divided between ciphers with and without a power consumption reduction. Third, the results obtained after inserting a short clock pulse and modifying the supply voltage of the standard Trivium cipher are presented. Finally, the results obtained after manipulating the control signal of the standard Trivium cipher with two different routings are presented.

### 5.1. Value of the Feedback Flip-Flops Transitions in the Attack Clock Cycles

It is noteworthy that, in a clock signal attack, the flip-flops most likely to fail are those whose input signals have a longer delay, i.e., those whose inputs are connected to a critical path. In the Trivium cipher, the flip-flops with the longest delay in their inputs should naturally be the first flip-flops of each of the three shift registers that perform the internal state of the cipher. We call these feedback flip-flops. As shown in Table 4, the feedback flip-flops are denoted by 0, 93 and 177. Table 4 shows, for each of the feedback positions, if, in the selected attack cycles, there is a change in the value of the flip-flops. Change is defined as the transition from 0 to 1 or from 1 to 0 in the clock cycle selected for the attack. With this analysis, we can see if it is possible to inject a fault into these flip-flops in each of the selected cycles, as, if the flip-flops do not change, is not possible to inject a fault. These transition values depend on the key and IV selected.

For example, considering Table 4 and clock cycle 1307, as there is no transition in the feedback flip-flops, it is not possible to inject faults. However, in clock cycle 1312, flip-flops 0 and 93 change; therefore, faults would tend to occur in those positions. This analysis is very interesting as it allows us to determine which are the next flip-flops in the internal register with the longest delay in their inputs.

### 5.2. Obtained Results Using Short Pulse Insertion and Nominal Supply Voltage

Table 5 and Table 6 show the results for the standard without power reduction Trivium versions and Table 7 and Table 8 show the results for the low-power Trivium versions. Table 5 and Table 7 show the number of fault injections achieved in each clock cycle for each pair of Trivium versions and Table 6 and Table 8 show the fault position in the internal register where the faults were injected. In all these tables, the clock cycles are those selected for the attacks and Triv. 1 and Triv. 2 are the two different routings of each version of the Trivium (e.g., Figure 2b). In Table 5 and Table 7, where the number is zero, no fault injections were injected; where the number is one, a successful attack was achieved with only one fault injection in the state register, while, where the number is greater than one, multiple faults were injected. In Table 6 and Table 8, the numbers represent the positions into the internal register where the faults were injected.

As shown in Table 5 and Table 7, when using our attack system, it is possible to inject only one faulty bit into most versions of the Trivium cipher. It is noteworthy that each pair of versions has different behaviors against the same attack, that is, the routing effect on the vulnerability when this type of attack is performed. In the case of the standard multi-radix ×2 Trivium, only multiple faults were injected. This is due to the fact that the clock pulse period must be selected more precisely. In spite of this, when carrying out a greater number of tests, by modifying the supply voltage, it was possible to inject only one faulty bit into the internal state as in the other ciphers.

On the other hand, if we consider Table 6 and Table 8, we can see that the fault positions that tend to appear are those located in the feedback positions or their neighbors. This is due to the fact that, if the delay in the inputs of the feedback flip-flops is greater due to the additional logic, it is more likely that these flip-flops are the first to fail. If we consider the standard Trivium in Table 4, it is possible to see that, in the clock cycles where the feedback positions change, for example, for clock cycle 1312, where the positions 0 and 93 change their values, the fault can be injected. On the contrary, if the feedback positions do not change, for example, for clock cycle 1307, the attacks are not able to inject any fault, as explained in the previous section. These results show that the selection of one or another pair of keys and IVs does not have an effect when using this technique as the fault is injected if, in the cycle selected for the attack, the feedback flip-flop has to change its value. In addition, it is always possible to change the clock cycle of the attack in order to make it match with the cycles in which value transitions occur in the feedback flip-flops.

In the case of the multi-radix and low-power versions, as shown in the tables, the fault positions are not only the feedback positions but also the intermediate and neighboring positions to the feedbacks. This is due to the great amount of logic added for the power reduction and for the multi-radix addition. The more logic added, the more critical paths and, therefore, more weak points where faults are injected.

### 5.3. Results Obtained by Modifying the Supply Voltage and the Pulse Clock Width in Standard Trivium

**Figure 14:**
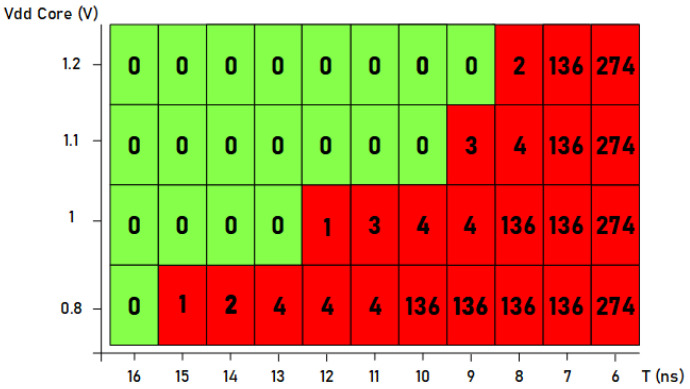
Number of faults injected into the standard Trivium cipher by modifying the supply voltage and the width of the clock pulse.

Figure 14 represents a plot with the number of faults produced when the supply voltage and the width of the induced clock pulse were changed. The supply voltage was varied from the minimum to the maximum (between 1.2 and 0.8 V) while changing the value of the period of the inserted clock pulse (between 6 and 16 ns). As it can be seen, with the maximum supply voltage, a smaller short pulse is necessary. For example, in the case of using 9 ns for the short pulse, no faults were injected; in the case of 8 ns, two faults were injected; and finally, in the case of 7 ns, a large number of faults were injected. In these cases, it can be seen that the difficulty in injecting faults was greater as there was a quick transition from injecting a small number of faults to a large number of faults. Therefore, in the case of using maximum supply voltage, the small pulse must be set between 8 and 9 ns to introduce only one faulty bit. On the contrary, if we consider the tests carried out using the minimum supply voltage of 0.8 V, not only it is possible to inject a single fault with a much larger short pulse size (15 ns), but also, if the size is modified to 11 ns, it is possible to inject a very low number of faults. Therefore, less precise control of the clock pulse width is required, which means that less precise test equipment is needed if the supply voltage is decreased.

### 5.4. Results Obtained by Manipulating the Control Signals in Standard Trivium

Table 9 and Table 10 show the results when the control signal was manipulated. In Table 9, the number of faults in each clock cycle is shown. Table 10 shows the positions of the faults represented in Table 9. The values of the change in the control signal were 15.051 and 16.050 ns, with the same change value, while the injected faults were different in each attack under the same conditions. Note that both tables present a total of five different tests, where the first three tests were performed using 15.051 ns and the last two using 16.050 ns. First, as shown in Table 9, it was possible to inject one faulty bit using this technique, for example, Test 2—15.051 ns in the attack clock cycle 1545 for Trivium 1—or Test 4—16.050 ns in the attack clock cycle 1545 for Trivium 2. Second, the number of injected faults changed suddenly from a few faults to a large number under the same conditions. Therefore, it is very sensitive to the variation of control signals. Third, if we consider the results shown in Table 10, the positions of the faults injected using this technique did not always correspond to the feedback positions, but also intermediate positions within the shift registers. These results are of great relevance, since, unlike the faults injected when using the short clock pulse technique, it was possible to inject faults both in the feedback flip-flops and in intermediate positions within the shift registers. Fourth, the results obtained show that there was a much greater influence of the routing of the ciphers; for example, for the attacks presented, if a change at 15.051 ns was used, the Trivium 1 cipher tended to fail, while, if a change of 16.050 ns was used, both ciphers tended to fail. These results show that the control signals were not routed with specific lines as in the case of the clock signals and, therefore, must be protected to prevent these vulnerabilities.

## 6. Conclusions

In this work, different methodologies for testing the vulnerabilities of different versions of the Trivium stream cipher implemented in ASIC against fault attacks are presented. We tested the standard and multi-radix ×2, ×8 and ×16 versions, as well as the low-power consumption versions of each of them. To test the vulnerabilities, two fault attack systems were designed and applied. The first one consisted of introducing short pulses in the clock input line combined with the modification of the supply voltage and circuit temperature. The second one consisted of manipulating the time at which the control signal of the circuit changed.

The results showed that, with both methodologies, it was possible to inject effective faults of one faulty bit into the internal state of each cipher. In the case of the short pulse introduction combined with the variation of the circuit temperature, in a range that did not produce damages in the circuit, it was observed that it had no effect on the possibility of injecting a greater or lower number of faults, which showed the same results for all temperatures. On the other hand, the results showed that key and IV selection had no effect on fault injection, since the same behavior was observed in the experiments performed for different keys and IVs and different attack clock cycles. However, it was observed that faults could only be injected into flip-flops that changed in that clock cycle. Therefore, the selection of the clock cycle affects the positions in which faults are injected. In the case of the short pulse introduction combined with supply voltage manipulation, the results showed that, if the value of supply voltage is the maximum or nominal, the difficulty of injecting faults is the same. On the contrary, if the supply voltage is the minimum, the periods of the required clock pulses are doubled (frequency needed ×0.5), considerably increasing the possibility of injecting faults. In the case of the control signal manipulation system, it was shown that it was possible to inject effective faults in different positions under the same conditions. Moreover, with this technique, not only it is possible to inject faults into the feedback registers, but also to inject them into the intermediate positions, with the additional information of injecting faults in both locations, which are all exploitable in DFA.

On the other hand, having two instances of the same Trivium allowed us to analyze the influence of routing on attacks. Although their response was slightly different and faults could be injected at different positions and times, the overall result was the same, since the vulnerability of the ciphers was maintained. In summary, the methodologies used and the attack systems designed allowed us to inject effective faults into all versions of the Trivium cipher, which were the faults injected into the critical paths, namely, the feedback positions, for the first methodology, and in any position, for the second one. Therefore, with these attack systems, it is possible to endanger the security of different versions of a Trivium cipher implemented in an ASIC, which is also extensible to NLFSR-based ciphers, for the first attack methodology, and to other ciphers, for the second methodology. This makes it necessary to introduce countermeasures against fault injection attacks through the clock signal or control signal in the ASIC implementations of these ciphers.

## Figures and Tables

**Table 1:** Values of the core power supply recommended by the manufacturer and those used for the test.

Power Supply	Max. (V)	Typ. (V)	Min. (V)
Recommended	1.32	1.2	1.08
Used	2.5	1.2	0.8

**Table 2:** Values of the circuit temperature recommended by the manufacturer and those used for the test.

Temperature	Max. (°C)	Typ. (°C)	Min. (°C)
Recommended	125	25	−40
Used	80	25	0

**Table 3:** Short-pulse range values obtained for each cipher in different scenarios. Case 1—nominal power supply. Case 2—minimum power supply. Case 3—nominal circuit temperature.

	Case 1 (ns)		Case 2 (ns)		Case 3 (ns)	
Cipher Version	Max.	Min.	Max.	Min.	Max.	Min.
Standard	7.5	6.95	16	12	7.5	6.95
Multi-radix ×2	8.92	6.92	22	15	8.92	6.92
Multi-radix ×8	7.1	7	14	11	7.1	7
Multi-radix ×16	9	7	21	18	9	7
Low power	8.8	6.9	16	14	8.8	6.9
Multi-radix ×2 LP	14.7	13	29	27	14.7	13
Multi-radix ×8 LP	9.8	7.4	21	17	9.8	7.4
Multi-radix ×16 LP	17.1	14.5	32.5	31.5	17.1	14.5

**Table 4:** Transition values of the standard Trivium feedback flip-flops.

Attack	Feedback Flip-Flops		
Clock Cycle	0	93	177
1307	No	No	No
1312	Yes	Yes	No
1351	No	No	Yes
1403	No	Yes	No
1545	Yes	Yes	Yes

**Table 5:** Number of faults injected into each pair of Trivium cipher versions in different clock cycles.

Trivium	Standard		Multi-Radix ×2		Multi-Radix ×8		Multi-Radix ×16	
Clock Cycle	Triv. 1	Triv. 2	Triv. 1	Triv. 2	Triv. 1	Triv. 2	Triv. 1	Triv. 2
1307	0	0	0	0	2	1	1	1
1312	2	1	146	146	2	1	2	0
1351	0	0	143	143	2	1	0	1
1403	1	0	136	136	0	1	1	1
1545	1	0	0	0	1	2	1	0

**Table 6:** Position of the faults injected into each pair of Trivium cipher versions in different clock cycles.

Trivium	Standard		Multi-Radix ×2		Multi-Radix ×8		Multi-Radix ×16	
Clock Cycle	Triv. 1	Triv. 2	Triv. 1	Triv. 2	Triv. 1	Triv. 2	Triv. 1	Triv. 2
1307	-	-	-	-	9/178	94	100	100
1312	0/93	0	*	*	93/97	97	104/107	-
1351	-	-	*	*	1/184	1	-	104
1403	93	-	*	*	-	98	120	101
1545	93	-	-	-	97	97/98	104	-

(-) no fault injected for this position, (*) too many faults injected.

**Table 7:** Number of faults injected into each pair of low-power Trivium cipher versions in different clock cycles.

Trivium	Standard LP		Multi-Radix ×2 LP		Multi-Radix ×8 LP		Multi-Radix ×16 LP	
Clock Cycle	Triv. 1	Triv. 2	Triv. 1	Triv. 2	Triv. 1	Triv. 2	Triv. 1	Triv. 2
1307	0	NA	2	0	0	1	2	2
1312	0	NA	2	1	2	1	1	1
1351	1	NA	0	0	1	1	1	1
1403	2	NA	1	1	0	0	2	1
1545	0	NA	0	0	1	0	0	2

(NA) data not available; the second cipher did not work correctly due to a manufacturing error.

**Table 8:** Position of the faults injected into each pair of low-power Trivium cipher versions in different clock cycles.

Trivium	Standard LP		Multi-Radix ×2 LP		Multi-Radix ×8 LP		Multi-Radix ×16 LP	
Clock Cycle	Triv. 1	Triv. 2	Triv. 1	Triv. 2	Triv. 1	Triv. 2	Triv. 1	Triv. 2
1307	-	-	0/1	-	-	4	1/4	103/104
1312	-	-	0/1	0	1/2	4	17	17
1351	93	-	-	-	1	1	17	17
1403	0/93	-	95	95	-	0	1/5	1
1545	-	-	-	-	0	0	-	19/25

(-) no fault injected for this position.

**Table 9:** Number of faults injected into the standard Trivium cipher when the control signals are changed.

Change (ns)	15.051 (Test 1)		15.051 (Test 2)		15.051 (Test 3)		16.050 (Test 4)		16.050 (Test 5)	
Clock Cycle	Triv. 1	Triv. 2	Triv. 1	Triv. 2	Triv. 1	Triv. 2	Triv. 1	Triv. 2	Triv. 1	Triv. 2
1312	2	0	10	0	1	0	149	0	149	6
1403	19	0	2	0	14	0	132	9	132	1
1545	10	0	1	0	10	0	136	1	136	6

**Table 10:** Position of the faults injected into the standard Trivium cipher when the control signals are changed.

Change (ns)	15.051 (Test 1)		15.051 (Test 2)		15.051 (Test 3)		16.050 (Test4)		16.050 (Test 5)	
Clock Cycle	Triv. 1	Triv. 2	Triv. 1	Triv. 2	Triv. 1	Triv. 2	Triv. 1	Triv. 2	Triv. 1	Triv. 2
1312	13/17	-	*	-	17	-	*	-	*	*
1403	*	-	17/73	-	*	-	*	*	*	74
1545	*	-	17	-	*	-	*	0	*	*

(-) no fault injected for this position, (*) too many faults injected.

## Data Availability

Not applicable.

## Abbreviations

The following abbreviations are used in this manuscript:

PA	power analysis
FA	fault analysis
IoT	Internet of Things
DFA	differential fault analysis
TSMC	Taiwan Semiconductor Manufacturing Company
NLFSR	nonlinear-feedback shift register
ASIC	application-specific integrated circuit
FPGA	field-programmable gate array
